# Maternal and fetal outcome of pregnancy related hypertension in Mettu Karl Referral Hospital, Ethiopia

**DOI:** 10.1186/s13048-015-0135-5

**Published:** 2015-03-15

**Authors:** Eshetu Seyom, Mubarek Abera, Million Tesfaye, Netsanet Fentahun

**Affiliations:** Department of Obstetrics/Gynecology and Surgery Coordinating Office, Jimma University, Jimma, Ethiopia; Department of Psychiatry, Jimma University, Jimma, Ethiopia; Department of Anesthesia, Jimma University, Jimma, Ethiopia; Department of Health Education and Behavioral Sciences, Jimma University, Jimma, Ethiopia

**Keywords:** Gestational, Hypertension, Preeclampsia, Eclampsia, Low resource country

## Abstract

**Background:**

Hypertensive disorders of pregnancy are the most common causes of adverse maternal & perinatal outcomes. Such investigations in resource limited settings would help to have great design strategies in preventing maternal and perinatal morbidity and mortality.

**Aim:**

To determine management outcome and factor associated with pregnancy related hypertensive disorder in Mettu Karl Referral Hospital, Mettu, Ethiopia.

**Method:**

A retrospective study deign was conducted at Mettu Karl Referral Hospital from 1^st^ January 2010 to December 1^st^ 2013 by reviewing medical records and logbooks. Descriptive, binary and multiple logistic regression analysis were used. A 95% CI and P- value of < 0.05 were considered statistically significant.

**Result:**

The magnitude of pregnancy related hypertensive disorder was 2.4%. Majority 82.6% of the mothers were in the age range between 18 to 34 year with a mean age and standard deviation (SD) of 24.4 (SD ± 5.12). Sever preeclampsia was the most prevalent diagnosis made to 35.5% of the mother, followed by 19% cases of eclampsia and 12.4% of HELLP. Fetal management outcomes indicates 120.37 perinatal mortality per 1000 deliveries and a stillbirth rate of 10.2%, low birth weight of 30.5%, and low APGAR score of 18.5%, abortion 10.7% and preterm delivery 31.4%.

**Conclusion:**

In this study severe preeclampsia is the most common of all pregnancy related hypertension disorders followed by Eclampsia. Fetal complications like low Apgar score and preterm deliveries were statistically significant and associated with fetal management outcomes.

## Introduction

Preeclampsia is a hypertensive disorder of pregnancy, which usually manifests after 20 weeks of gestation with hypertension and proteinuria,while hypertension being defined with blood pressure at least 140 mm Hg for systolic and/or 90 mm Hg for diastolic on at least two occasions and at least 4—6 hrs apart in women known to be normotensive beforehand [1-3]. Severe hypertension is considered if sustained rises in blood pressure to the level of > = 160 mm Hg for systolic), and/or 110 mm Hg for diastolic, [1,3,4]. When convulsions occur in addition to these signs, of preeclampsia the condition is referred to as eclampsia [5].

Preeclampsia is primarily a disorder of nulliparous, but multiparous pregnant women with a new partner have an elevated risk of preeclampsia similar to that of nulliparous women [6]. Delivery of placenta is the only treatment yet known, indicating placenta is the primary sponsor to the pathogenesis of preeclampsia [7].

Following the drama of hypertensive disorders during pregnancy, 12–22% of all pregnancies, have a tragic story [8,9]. Increased mortality and morbidity as a result of Preeclampsia during pregnancy is not only a terror to the mother but also to the fetus [10]. Preeclampsia has hang around till this century and continues being public health concern in both developed and developing countries [11]. The usual markers of developing countries like poor antenatal care, illiteracy, lack of awareness and poverty in developing countries continues to favor these nightmares of pregnant mothers [8]. Thus, the aim of this study is to highlight the management outcome and factor associated with pregnancy related hypertensive disorder in an Ethiopian hospital.

## Materials and methods

### Study setting and period

This study was conducted from 1^st^ January 2010 to December 1^st^ 2013 in Mettu karl hospital. The hospital has 138 health professionals in different disciplines. The number of pregnant mother in the catchment per year is 55,860. There are a total of 160 beds in all wards of which 47 beds (38 gynacology and 9 in obstetrics) and three delivery coaches.

### Study design and participants

A three years hospital based retrospective study design was implemented to determine management outcome and factor associated with pregnancy related hypertensive disorders. All pregnant mothers who gave birth at Metu karl hospital over the study period (three years) were included for the review.

### Instruments and data collection methods

The data for this study was obtained from admission registration logbooks, delivery registration books and patient charts. The data was collected using a pre-tested check list questioner which was a socio-demographic variables, obstetrics history, sign and symptoms at presentation, laboratory result, maternal and fetal outcomes. The data collectors were given two days training on how to review and abstract the required and pertinent information from the main document. Data collection tools (the checklists) to collect the data from the registration books and client cards was prepared and pretested with similar group as the target group and excluded from the sample. To assure the quality of the data, data collectors was supervised by principal investigator every day.

### Ethical statement

Ethical approval is obtained from Ethical review board of Jimma University, College of Public Health & Medical Science. Metu karl Hospital gives permission to conduct the study. Then, study participants had also gave informed written consent. The privacy of mothers was kept confidential.

### Data analysis

The collected data were manually checked for completeness and for any inconsistency then coded and entered into SPSS version 16.0 for data processing and analysis. Descriptive, binary and multiple logistic regression analysis were used. On binary logistic regression analysis, a p-value ≤ 0.25 was used as a candidate for multiple logistic regression analysis. A 95% CI and P- value of <0.05 was considered statistically significant and crude/adjusted odds ratio calculated.

## Result

### Socio demographic characteristics

A total of 5415 pregnant mothers admitted to Mettu karl obstetrics ward for delivery from January 1^st^ 2010- December 30/2013 and their record was reviewed of which 130 mothers were found to have the diagnosis of pregnancy related hypertensive disorder. Among these 121 (93%) mothers who have a record of HDP were retrieved for further analysis. The minimum and maximum age of the mother were 15 and 39 respectively with a mean and standard deviation of 24.4 (SD ± 5.12). The majority 100 (82.6%) of the mothers were in the age range between 18 to 34 years. As to their residence and background information, 84 (69.8%) were from outside of Mettu town, 62 (51.2%) un-booked for antenatal care follow up, and 63 (52.1%) were primiparous.

Majority 58 (47.9%) of the mother presented to the Hospital with a chief complain of headache. Nearly 87 (71.9%) of the mother had maximum blood pressure record of BP ≥ 16o/110. Sever preeclampsia is the most common hypertensive disorder of pregnancy accounting 35.5%, followed by eclampsia which contributed 19%, mild preeclampsia 14.9%, HELLP syndrome 12.4%, gestational HTN 13.2%, and chronic HTN 4.1% and two patients were superimposed preeclampsia Table 1.

**Table 1:** **Perinatal outcome of mothers with hypertensive disorder of pregnancy**

**Perinatal outcome**	**Type of hypertension (Hypertensive disorder of pregnancy)**					
	**Mild preeclampsia**	**Sever preeclampsia**	**HELLP**	**Eclampsia**	**Gestational**	**Chronic**
	**No (%)**	**No (%)**	**No (%)**	**No (%)**	**No (%)**	**No (%)**
**Fetal weight (grams)**						
Wt. ≥ 2500	13 (72.2)	26 (60.5)	3 (20.0)	15 (65.2)	14 (87.5)	4 (66.7)
Wt. = 1000-2499	5 (27.8)	12 (27.9)	9 (60.0)	6 (26.1)	1 (6.2)	0 (0.0)
Fetal outcome						
Stil birth	2 (11.1)	6 (14.0)	3 (20.0)	0 (0.0)	0 (0.0)	0 (0.0)
Low APGAR/<7/	2 (11.1)	7 (16.7)	4 (26.7)	3 (13.0)	3 (18.8)	1 (16.7)
**Abortion**	0 (0.0)	5 (11.6)	3 (20.0)	2 (8.7)	1 (6.2)	2 (33.3)
**Neonatal outcome**						
**Early neonatal**						
**Death**	0 (0.0)	1 (2.3)	0 (0.0)	0 (0.0)	1 (6.2)	0 (0.0)

HELLP-Hemolytic Elevated Liver enzyme and low platelet.

**Figure 1:**
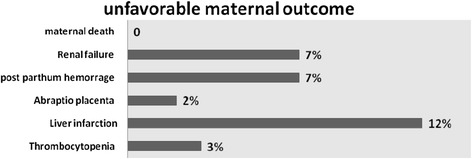
**Percentage of mothers with unfovorable outcome (n** = **121).**

There were eight mothers with HDP complicated with renal failure (6.6%), nine (7.4%) with postpartum hemorrhage and two with abruptio placenta and there was no maternal death during the study period (Figure 1).

**Figure 2:**
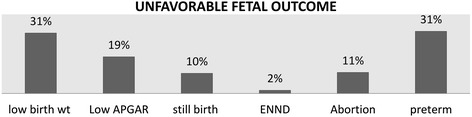
**Percentage of unfavorable fetal outcome (n** = **108).** ENND-Early Neonatal Death.

In this study of all 108 mothers with unfavorable fetal outcomes, the perinatal mortality rate was 120.37 per thousand deliveries and 11 still births yielding the still birthrate of 10.185%. The rate of low and very low birth weight were 24.8% and 3.3% respectively and there were 20 (18.5%) low APGAR score and preterm delivery 34 (28.1%) among 108 deliveries .there were 2 early neonatal death and 13 (10.7%) abortion (Figure 2).

As to the management of mothers, 2 (1.7%) of them were managed conservatively, 38 (31.4%) with anti-hypertensive, and 81 (66.9%) were given both anti-hypertensive and anti convulsant medications. On the other hand, onset of labor was spontaneous for 60 (49.6%) of the mother, and induction was made to 58 (47.9%) while elective cesarean delivery was made to 3 (2.5%) of the mother. Majority of the mothers delivered with spontaneous vaginal delivery 89 (73.6%), cesarean delivery 21 (17.4%), forceps/vacuum 9 (7.4%) and two destructive delivery (Table 2).

**Table 2:** **Onset of labor and intervension of mothers with hypertensive disorder of pregnancy**

**Variables**	**Type of hypertension (hypertensive disorder of pregnancy)**			
	**Preeclampsia**	**Eclampsia**	**Gestational**	**Chronic**
Onset of labor				
Spontaneous	33 (55)	15 (25)	10 (16.7)	2 (3.3)
Induction	41 (70.7)	8 (13.8)	5 (8.6)	4 (6.9)
Elective Cesarean	2 (66.7)	0 (0)	1 (33.3)	0. (0)
Mode of delivery				
Vaginal	56 (62.9)	19 (21.3)	9 (10.1)	5 (5.6)
Caesarean section	11 (52.4)	2 (9.5%)	7 (33.3)	1 (4.8)
Forceps/vacuum	7 (77.8)	2 (22.2)	0 (0)	0 (0)
Destructive	2 (100)	0 (0)	0 (0)	0 (0)
How patients managed				
Conservatively	0 (0)	0 (0)	2 (100)	0 (0)
Antihypertensive	17 (44)	2 (5.3)	14 (36.8)	5 (13.2)
Antihypertensive + anticonvulsant	59 (72.8)	21 (25.9)	0 (0)	1 (1.2)

### Predictors of maternal management outcomes of HDP

Five of the variables were found to be candidates in the binary logistic analysis for the final model. Therefore, a multivariate approach was applied to determine which factors best explained and predict maternal management outcome.

The outcome of the final multiple logistic regression models indicated that address, gestational age and length of hospital stay dropped from the final model. In this analysis maximum blood pressure record has significant statistical association with maternal management outcome of HDP (AOR = 26, 95% CI: 5.11-132.35, p-value = 0.001). The presence of albumin in the urine has statistical association with maternal management outcomes of HDP (AOR = 37.15, 95% CI: 6.58-209.85, p-value = 0.001. Mothers who had Proteinuria were more likely to develop unfavorable outcome as compared with mothers with negative Proteinuria (Table 3).

**Table 3:** **Binary logistic regression on factors associated with material management outcomes**

**Variables**	**Management outcomes**		**COR (95% CI)**
**Address**	**Favorable**	**Un-favorable**	
**Metu**	12	25	1
**Out metu**	14	70	2.40 (.98-5.88)
**Parity**			
**Multipara**	12	46	1
**Nullipara**	142	46	0.91 (0.38-2.18)
**Antenatal care**			
**Yes**	13	45	0.68 (0.28-1.64)
**No**	13	50	1
**Gestational age**			
**Term**	20	55	1
**Preterm**	5	29	2.11 (0.72-6.20)
** Abortion**	1	11	4.00 (0.49-33.00)
**Maximum BP**			
**140/90**-**159/109 mm Hg**	18	16	1
**>160/110 mm Hg**	8	79	11.11 (4.12-29.93)*
**Urine albumin**			
**Negative**	14	7	1
**Positive**	46	54	10.88 (3.78-31.27)*
**Length of hospital stay**			
**<7**	7	8	1
**>or** = **7**	13	52	2.03 (.75-5.54)

*Significant at p-value ≤ 0.001 is reference logical reference.

-BP: blood presusure.

### Predictors of fetal management outcomes of HDP

Seven of the variables were found to be significantly associated in the binary logistic analysis. Therefore, a multivariate approach was applied to determine which factors best explained and predict maternal management outcome. The outcome of the final stepwise multiple logistic regression models indicated that address, antenatal care, maximum blood pressure record, urine albumin, and length of hospital stay dropped from the final model.

In this analysis gestational age has significant statistical association with fetal management outcome of HDP (AOR = 80.4, 95% CI: 12.68-509.67, p-value <0.001). Preterm newborns are more likely to develop unfavorable fetal outcome as compared with term. APGAR score has significant statistical association with fetal management outcome of HDP (AOR = 22.96, 95% CI: 3.08-171.21, p-value = 0.002). Newborns with low APGAR score are more likely to develop unfavorable fetal outcome as compared with newborns with good APGAR score (Table 4)

**Table 4:** **Bivariate on factors associated with fetal management outcomes**

**Variables**	**Management outcomes**		**COR (95% CI)**
	**Favorable**	**Un-Favorable**	
**Address**			
**Metu**	22	15	1
**Out metu**	38	46	1.81 (0.82-3.98)
**Parity**			
**Multipara**	29	29	1
**Nullipara**	31	32	0.97 (0.48-1.99)
**Gestational age**			
**Term**	60	15	1
**Preterm**	3	31	41.33 (11.12-153.68)*
**Antenatal care**			
**Yes**	32	26	0.34 (0.16-0.73)
**No**	28	35	1
**Maximum BP**			
**140/90**-**159/109 mm Hg**	22	12	1
**>160/110 mm Hg**	38	49	2.06 (0.91-4.67)
**Albimin**			
**Negative**	14	7	1
**Positive**	46	54	2.08(0.78-5.59)
**APGAR**			
**>or** = **7**	60	29	1
**<7**	3	17	11.72 (3.18-43.23)*
**Length of hospital stay**			
**<7 days**	47	32	1
**>7 days**	16	26	2.39 (1.11-5.14)

*Significant at p-value ≤ 0.05 1 is reference logical reference.

-BP: blood pressure.

## Discussion

Geographic regions of the world reflect various prevalence of hypertensive disorder of pregnancy, from the lowest 1.5% in Sweden to 7.5% in Brazil [12,13]. In developing countries the pooled incidence of preeclampsia is 3.4% [14]. However recent analysis of large database from 29 countries, estimates the global distribution of the incidence of all deliveries to be 0.29%, 2.16% and 0.28% for chronic hypertension, pre-eclampsia and eclampsia respectively [15].

The magnitude of pregnancy related hypertensive disorder in our study was 2.4%. Severe preeclampsia was common in 35.5% of pregnant mothers and followed by eclampsia. This is not surprising giving the fact that most of the mothers were young ages and with low socioeconomic status. This has been widely investigated in the past decade and now a days being primigravida, living in low socio economic status and young ages are the characteristics of hypertensive disorder of pregnancy [12,16-18]. Placental abruption was as low as 2% in our case compared to some studies who reported as high as 20% [19], this might be explained to adequate blood pressure monitoring as no maternal death or intracranial hemorrhage is recorded. In line with many other studies [20], 71.9% of our mothers had maximum blood pressure record which read ≥16o/110 and HELLP syndrome in 12.4% of the cases.

A multivariate approach was applied to determine which factors best explained and predict maternal management outcome. The outcome of the final stepwise multiple logistic regression models indicated that address, antenatal care, maximum blood pressure record, urine albumin, and length of hospital stay dropped from the final model. Other studies also reflected socio-economic status and antenatal care provision plays the same role as biochemical markers of preeclampsia like urine albumin [19,21,22].

Concerning with the management of our patients the hospital being well equipped and well- staffed and giving service for 24 hours, early presentation of the mothers to the hospital and early intervention might contribute to no maternal death. In developed countries pregnancy related acute renal failure has decreased, current estimates are around 1–2.8% [23], where as in developing countries it is higher and responsible for both maternal and fetal morbidity and mortality [20]. Some setups showed an alarmingly high values to a 36% [24]. Our investigation indicated the incidence to be 6.6%, which is relatively low this might be due to liberalization with abortion laws, improved health care facilities and more effective measures of care full prevention. On top of this Mettu Karl hospital is well staffed and well equipped hospital compared to other national hospitals in the country.

In our analysis maximum blood pressure record has significant statistical association with maternal management outcome of HDP (AOR = 26, 95% CI: 5.11-132.35, p-value <0.001). The presence of albumin in the urine has statistical association with maternal management outcomes of HDP (AOR = 37.15, 95% CI: 6.58-209.85, p-value <0.001. Mothers who had Proteinuria were more likely to develop unfavorable outcome as compared with mothers with negative Proteinuria.

Additionally fetal outcome was affected by gestational age with significant statistical association (AOR = 80.4, 95% CI: 12.68-509.67, p-value <0.001) as well with the Apgar score (AOR = 22.96, 95% CI: 3.08-171.21, p-value = 0.002). Preterm newborns are more likely to develop unfavorable fetal outcome as compared with term ones. APGAR score has significant statistical association with fetal management outcome of HDP. Newborns with low Apgar score are more likely to develop unfavorable fetal outcome as compared with newborns with good Apgar score.

In conclusion we say both maternal and fetal outcome is highly dependent on socio-economic status, gestational age and maximum blood pressure of the mother. We recommend effective and competent health service provision through antenatal care and close follow up and management with available resources could improve mortality and morbidity as shown here with low frequencies of complications like renal failure, HELLP syndrome, placental abruption and no maternal death in this referral hospital with a huge catchment area.

## Abbreviations

HELLP: Hemolysis elevated liver enzyme, and low platlate
APGAR: Appearance, pulse, grimace, activity and reflex
BP: Blood Pressure
HDP: Hypertensive disorder of pregnancy
MmHg: Millimeter mercury
